# Tea leaf disease and insect identification based on improved MobileNetV3

**DOI:** 10.3389/fpls.2024.1459292

**Published:** 2024-09-27

**Authors:** Yang Li, Yuheng Lu, Haoyang Liu, Jiahe Bai, Chen Yang, Haiyan Yuan, Xin Li, Qiang Xiao

**Affiliations:** ^1^ Key Laboratory of Tea Quality and Safety Control, Ministry of Agriculture and Rural Affairs, Tea Research Institute, Chinese Academy of Agricultural Sciences, Hangzhou, China; ^2^ Hangzhou Ruikun Technology Co., Ltd., Hangzhou, China; ^3^ Tea Station of Xinchang County, Shaoxing, China

**Keywords:** tea leaf diseases and insects, convolution neural network, transfer learning, MobileNetV3, recognition and classification

## Abstract

Accurate detection of tea leaf diseases and insects is crucial for their scientific and effective prevention and control, essential for ensuring the quality and yield of tea. Traditional methods for identifying tea leaf diseases and insects primarily rely on professional technicians, which are difficult to apply in various scenarios. This study proposes a recognition method for tea leaf diseases and insects based on improved MobileNetV3. Initially, a dataset containing images of 17 different types of tea leaf diseases and insects was curated, with data augmentation techniques utilized to broaden recognition scenarios. Subsequently, the network structure of MobileNetV3 was enhanced by integrating the CA (coordinate attention) module to improve the perception of location information. Moreover, a fine-tuning transfer learning strategy was employed to optimize model training and accelerate convergence. Experimental results on the constructed dataset reveal that the initial recognition accuracy of MobileNetV3 is 94.45%, with an F1-score of 94.12%. Without transfer learning, the recognition accuracy of MobileNetV3-CA reaches 94.58%, while with transfer learning, it reaches 95.88%. Through comparative experiments, this study compares the improved algorithm with the original MobileNetV3 model and other classical image classification models (ResNet18, AlexNet, VGG16, SqueezeNet, and ShuffleNetV2). The findings show that MobileNetV3-CA based on transfer learning achieves higher accuracy in identifying tea leaf diseases and insects. Finally, a tea diseases and insects identification application was developed based on this model. The model showed strong robustness and could provide a reliable reference for intelligent diagnosis of tea diseases and insects.

## 1 Introduction

Tea is a significant economic crop in China, characterized by extensive cultivation and a wide variety of cultivars (Li et al., 2022). However, during its growth and cultivation, tea is susceptible to pests and diseases, which directly impact its quality and quantity (Liu and Wang, 2021). Tea production is reduced by about 20% each year due to tea leaf diseases and insects (Xue et al., 2023).The traditional approach to identifying pests and diseases in tea plants has relied heavily on the experience and visual inspection of technicians. However, this methodology is often constrained by the lack of specialized personnel and insufficient timeliness in the identification process (Huang et al., 2019). Consequently, the real-time and efficient monitoring of pest and disease conditions in tea plantations is crucial for precise pest management and the assurance of tea quality and safety.

With the advancement of machine vision technology, image processing and machine learning methods have been widely utilized in the identification of crop pests and diseases (Pal and Kumar, 2023; Pan et al., 2022). Sun et al. (2019) proposed an algorithm that combines simple linear iterative clustering with Support Vector Machine (SVM). This method effectively extracts important tea disease patterns from complex backgrounds, laying a solid foundation for further research on tea disease identification. Yousef et al. (2022) developed an apple disease recognition model using digital image processing and sparse coding, achieving an average accuracy rate of 85%. However, most of these studies are based on the identification of insects and diseases using characteristics such as color, texture, and shape, which often rely on manual selection and design. This dependency limits the adaptability of models to the environment, consequently resulting in weaker accuracy and universality in pest and disease classification.

In response to the problems of machine learning methods, more and more scholars are using deep learning models to identify tea leaf disease and insect (Chen et al., 2020; Qi et al., 2022; Liu and Zhang, 2023; Zhang and Zhang, 2023). Various models, including AlexNet, GoogLeNet, VGG, and ResNet (Krizhevsky et al., 2012; Szegedy et al., 2015; Simonyan and Zisserman, 2015), have demonstrated outstanding performance in crop disease identification. For example, an optimized Dense Convolutional Neural Network structure, presented by Waheed et al. (2020), achieved 98.06% accuracy in classifying corn leaf diseases. Li et al. (2022) integrated the SENet module into the DenseNet framework for tea disease identification using transfer learning. Sun et al. (2023) introduced TeaDiseaseNet based on YOLOv5 for detecting six tea leaf diseases, despite lower recognition rates and high-resolution image challenges. The above recognition models are improved by using a large-scale CNN, which is more computationally complex, has a slightly larger number of parameters, and imposes high requirements for deployment and application. Therefore, a lightweight recognition model with high accuracy is more valuable in actual production.

In the application of lightweight neural networks, MobileNet is often used as a basic model (Ullah et al., 2023; Peng and Li, 2023). To reduce the number of model parameters and the amount of computation, making the model more lightweight while ensuring good recognition results, researchers have improved the model structure and embedded an attention mechanism (Chen et al., 2022; Bi et al., 2022). However, in the identification of tea diseases, due to the small size of the dataset and the relatively sparse distribution of disease spots in some images, existing lightweight models struggle to achieve high-precision classification results for this problem.

Therefore, this study addresses the aforementioned issues by proposing a network model named MobileNetV3-CA, which integrates MobileNetV3 (Howard et al., 2019) with a Coordinate Attention (CA) module (Hou et al., 2021). The CA module enhances the model’s discriminative ability by expanding the local receptive field through the incorporation of attention mechanisms. Given the limited research on tea leaf diseases compared to fruit and cereal crops (Mu et al., 2023), images of tea leaf diseases were collected and augmented to construct a dataset containing 17 common types of tea leaf diseases and insects. Utilizing transfer learning, the model was pre-trained on a large-scale public dataset and then fine-tuned on a dataset of tea leaf diseases and insects to accelerate convergence and improve accuracy and robustness with limited samples. Finally, the effectiveness of the MobileNetV3-CA network model was validated through the recognition of common tea leaf diseases and insects, as well as testing within application programs.

## 2 Materials and methods

### 2.1 Construction of image data set of tea leaf diseases and insects

**Figure 1:**
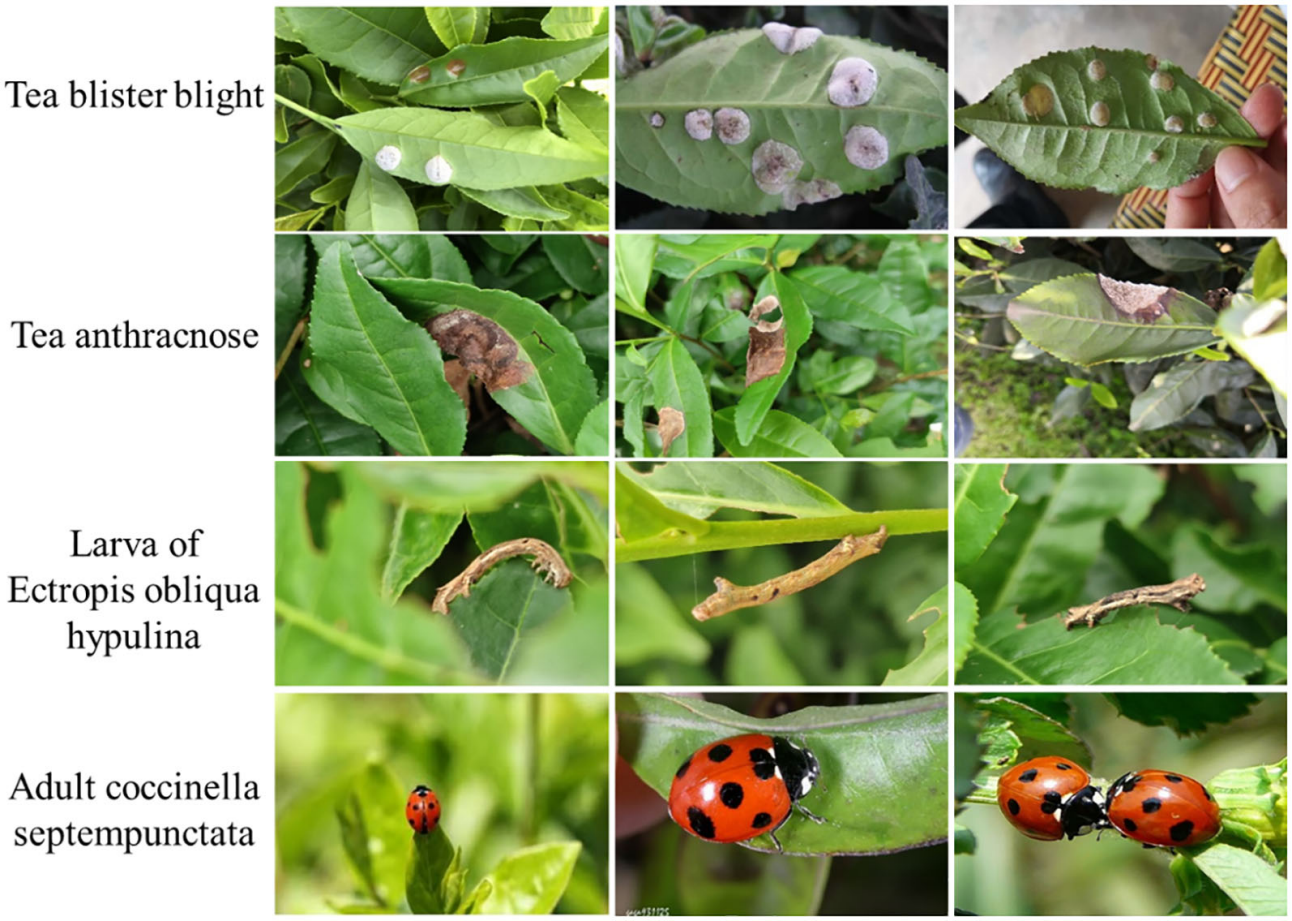
Some image samples of tea leaf diseases and insects in the data set.

**Figure 2:**
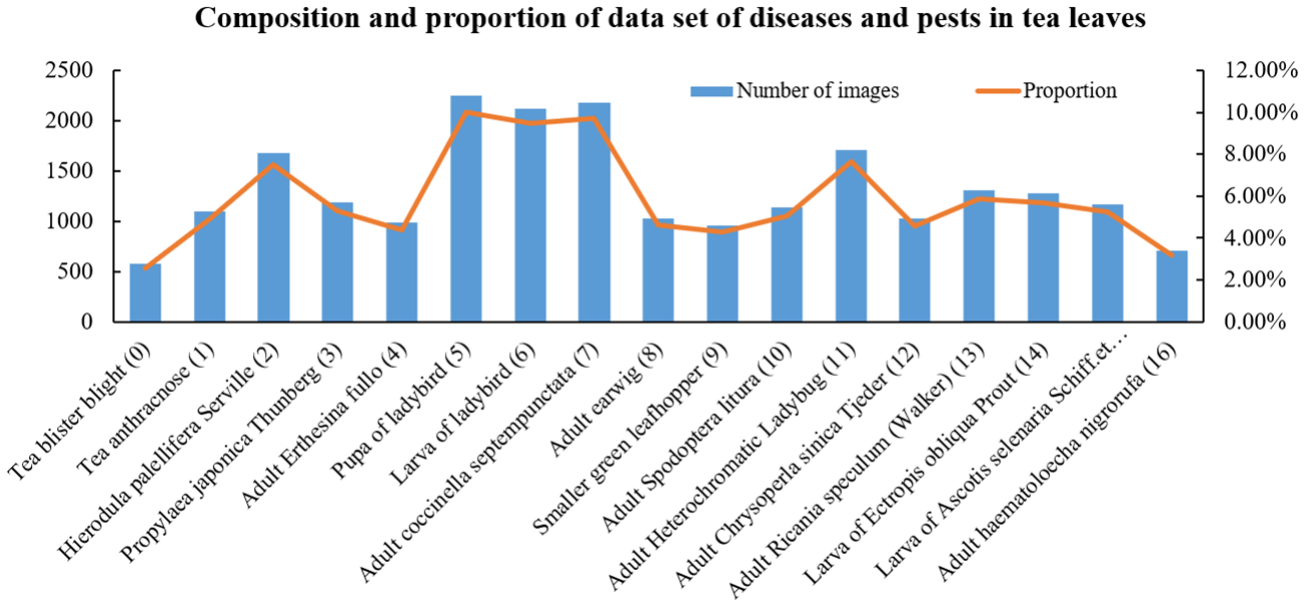
Proportion and quantity of all kinds of diseases and insect pests in the original data set.

The images of tea leaf diseases and insects used in this study were sourced from tea-producing regions in Xinchang County, Zhejiang Province. The primary tea plant varieties involved are Longjing 43, Wuniuzao, Jiukeng, and Zhongcha 108. Data collection occurred from March to April each year, from 2021 to 2023, during the high incidence period of tea diseases and insects, facilitating the comprehensive collection of disease and insect data. This study employed various smartphones, including models from brands such as Huawei, Xiaomi, and Apple, for image capture. The majority of image data was acquired through the team-developed backend of the “Xinchang Tea Guardian” WeChat mini-program. In total, 22,380 images were collected, encompassing 17 types of tea leaf diseases and insects, such as anthracnose, tea blister blight, ectropis obliqua hypulina. Some image samples of tea leaf diseases and insects in the data set are shown in **Figure 1**. Considering the significant morphological differences among certain insects in their larval, pupal, and adult stages, this study categorizes them into distinct groups. For instance, ladybugs and their larvae, as well as corn earworm larvae and adults, are included in separate categories. Furthermore, due to the uncertainty in collecting crop disease images, the distribution of tea leaf disease and insect images obtained is highly uneven. The number and proportion of various tea diseases and insects are shown in **Figure 2**. For instance, tea blister blight accounts for approximately 3% of the total, while ladybird pupa images constitute around 11%.

### 2.2 Data preprocessing

**Figure 3:**
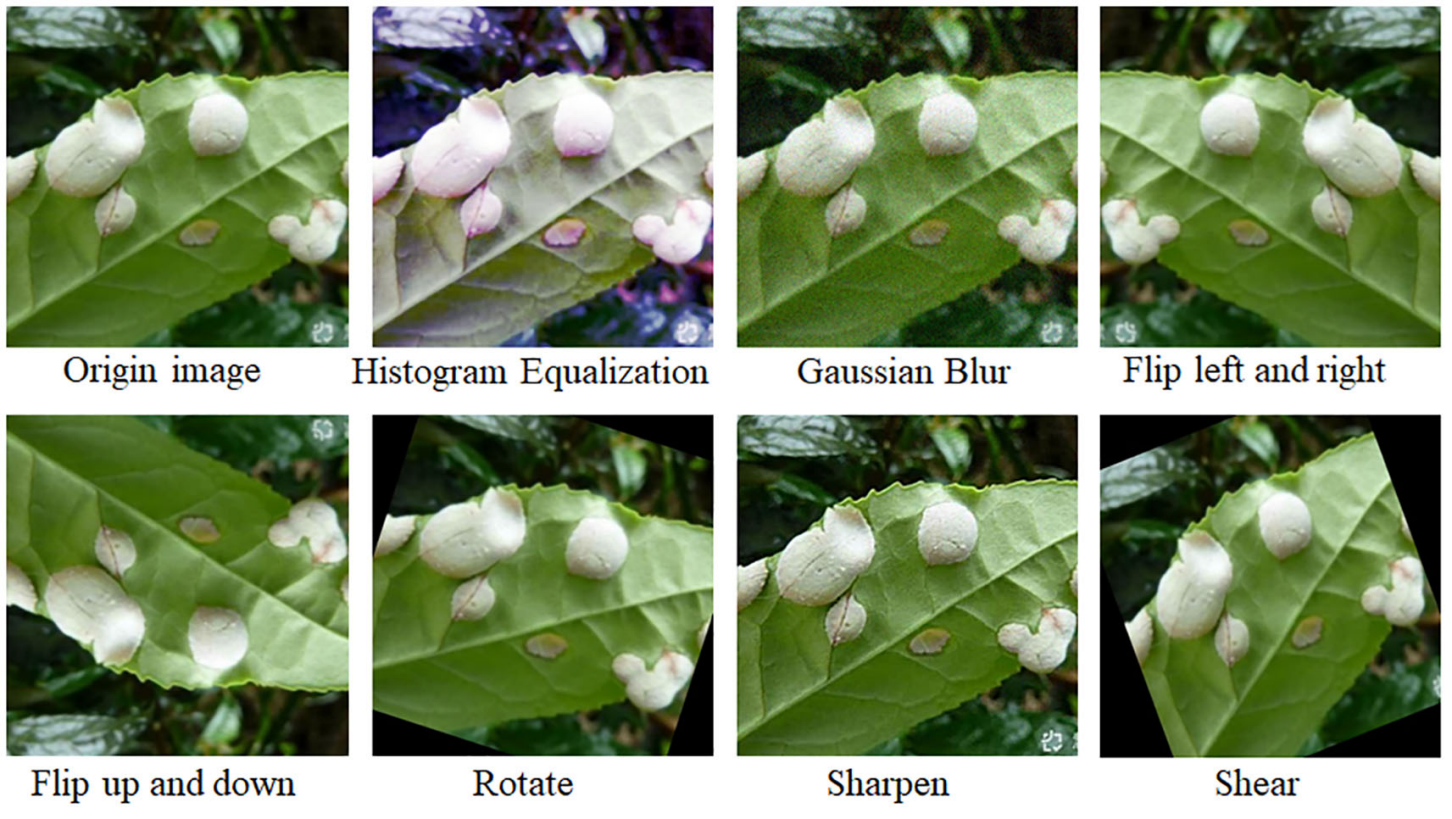
Examples of data augmentation.

Based on the distribution of the statistical samples shown in **Figure 2**, the number of images for different categories of diseases and insects ranges from 500 to 2000. To prevent the model from overfitting due to insufficient training samples, data augmentation techniques were employed. These operations include flipping, adding Gaussian noise, adjusting contrast, rotation, shear, and adding histogram equalization, all of which were applied in random order. Flipping refers to randomly flipping pictures up and down or left and right with a probability of 0.5. Adding Gaussian noise refers to applying Gaussian noise to images with a probability of 0.5, using a Gaussian kernel with a random standard deviation sampled uniformly from the interval [0.0, 0.6]. Adjusting contrast refers to modifying the contrast of images according to 127 + alpha*(*v*-127), where *v* is a pixel value and alpha is sampled uniformly from the interval [0.75, 1.5] (once per image). Rotation refers to rotating images by -20 to 20 degrees with a probability of 0.5. Shear refers to shearing images by -20 to 20 degrees with a probability of 0.5. Adding histogram equalization refers to applying histogram equalization to input images with a probability of 0.5. Data augmentation could enhance sample diversity and simulate natural conditions for the identification of diseases and insects. After data augmentation, the number of images for each category was increased to 2000, totaling 34,000 images. For example, the data augmentation process for tea blister blight is illustrated in **Figure 3**. The augmentation adjusted the original images’ rotation angle, brightness, and blurriness, highlighting the local details of diseases and insects. Additionally, considering the potential differences in image format and size due to varying sample sources, all images were standardized to a uniform size of 224 pixels × 224 pixels.

### 2.3 Construction and improvement of disease and insect recognition model

#### 2.3.1 An overall introduction to the improved model

Common lightweight neural network models include SqueezeNet (Iandola et al., 2016), MobileNet, EfficientNet (Tan and Le, 2019), among others. In this study, MobileNetV3 from the MobileNet series was selected. The MobileNetV3 model retains its lightweight characteristics while continuing to utilize the depthwise separable convolutions and inverted residual modules from the MobileNetV2 model (Sandler et al., 2018). It enhances the bottleneck structure by integrating the SE (Squeeze-and-Excitation) module (Hu et al., 2018), which strengthens the emphasis on significant features and suppresses less important ones. Additionally, the new hard-swish activation function is adopted to further optimize the network structure. These improvements allow the MobileNetV3 model to maintain its lightweight nature while enhancing accuracy in tasks such as image classification. The MobileNetV3 model is available in large and small versions based on resource availability, and this study employs the MobileNetV3-large model as the baseline. Its structural parameters are shown in **Table 1**.

**Table 1:** MobileNetV3-large structure.

Input	Operation	SE module	Activation function	Stride
224^2^×3	Conv2d	–	HS	2
122^2^×16	Bneck, 3×3	–	RE	1
122^2^×16	Bneck, 3×3	–	RE	2
56^2^×24	Bneck, 3×3	–	RE	1
56^2^×24	Bneck, 5×5	√	RE	2
28^2^×40	Bneck, 5×5	√	RE	1
28^2^×40	Bneck, 5×5	√	RE	1
28^2^×40	Bneck, 3×3	–	HS	2
14^2^×80	Bneck, 3×3	–	HS	1
14^2^×80	Bneck, 3×3	–	HS	1
14^2^×80	Bneck, 3×3	–	HS	1
14^2^×80	Bneck, 3×3	√	HS	1
14^2^×112	Bneck, 3×3	√	HS	1
14^2^×112	Bneck, 5×5	√	HS	2
7^2^×160	Bneck, 5×5	√	HS	1
7^2^×160	Bneck, 5×5	√	HS	1
7^2^×160	Conv2d, 1×1	–	HS	1
7^2^×960	Pool, 7×7	–	–	1
1^2^×960	Conv2d, 1×1,NBN	–	HS	1
1^2^×17	Conv2d, 1×1,NBN	–	–	1

Conv2d stands for convolution layer; Bneck stands for a bottleneck structure; Pool represents pooling layer; NBN stands for batch normalization; RE and HS stand for ReLU6 and hard-swish, respectively.

#### 2.3.2 Improvement of attention mechanisms

**Figure 4:**
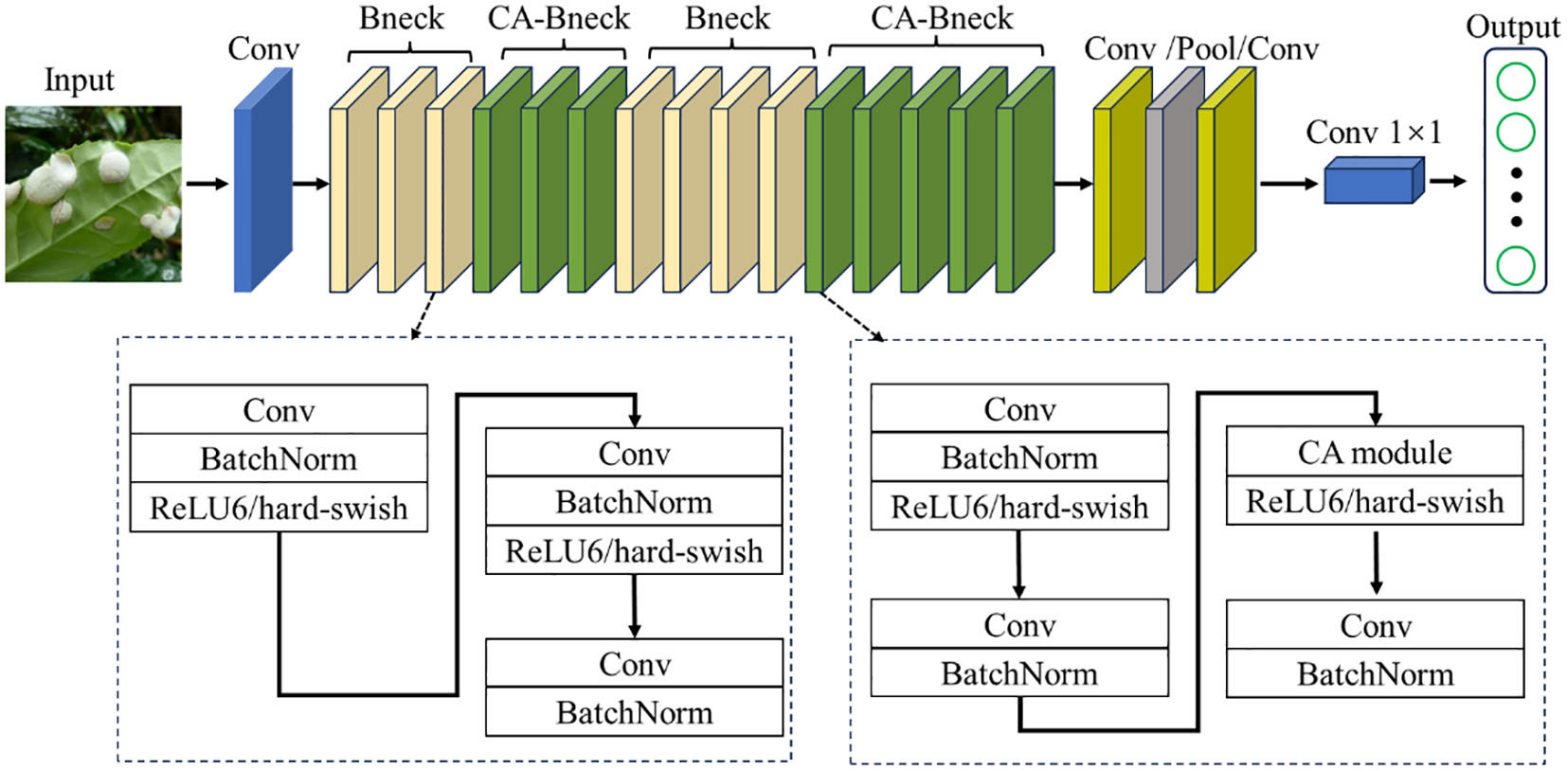
The structure of MobileNetV3-CA model. Conv stands for convolution layer; Bneck and CA-Bneck stand for a bottleneck structure and a bottleneck structure after introducing coordinate attention module, respectively; Pool represents pooling layer; BatchNorm stands for batch normalization; ReLU6/hard-swish stand for activation function.

While incorporating the SE module into the bottleneck structure of MobileNetV3-Large has indeed improved model performance, the SE module only considers information between channels to determine the importance of each channel. However, it overlooks the crucial positional information in the visual space. As a result, the model can only capture local feature information, leading to issues such as scattered regions of interest and limited performance. To address these limitations, the ECA module improves on the SE module by avoiding dimensionality reduction and capturing cross-channel interaction information more efficiently (Wang et al., 2020). Even though the ECA module is an improvement over the SE module, it still only considers the information between channels in essence (Jia et al., 2022). Therefore, in order to improve the recognition rate of the model and enhance its ability to capture the location information of tea leaf diseases and insects, the coordinate information must be considered. In this study, we replace the SE module in the MobileNetV3 structure with the CA module to improve MobileNetV3. The overall structure of the improved MobileNetV3-CA model is shown in **Figure 4**. To accurately obtain the relative position information in the image of diseases and insect pests of tea leaves, the CA module was introduced into the attention module of the bottleneck structure of layers 4-6 and layers 12-16.

**Figure 5:**
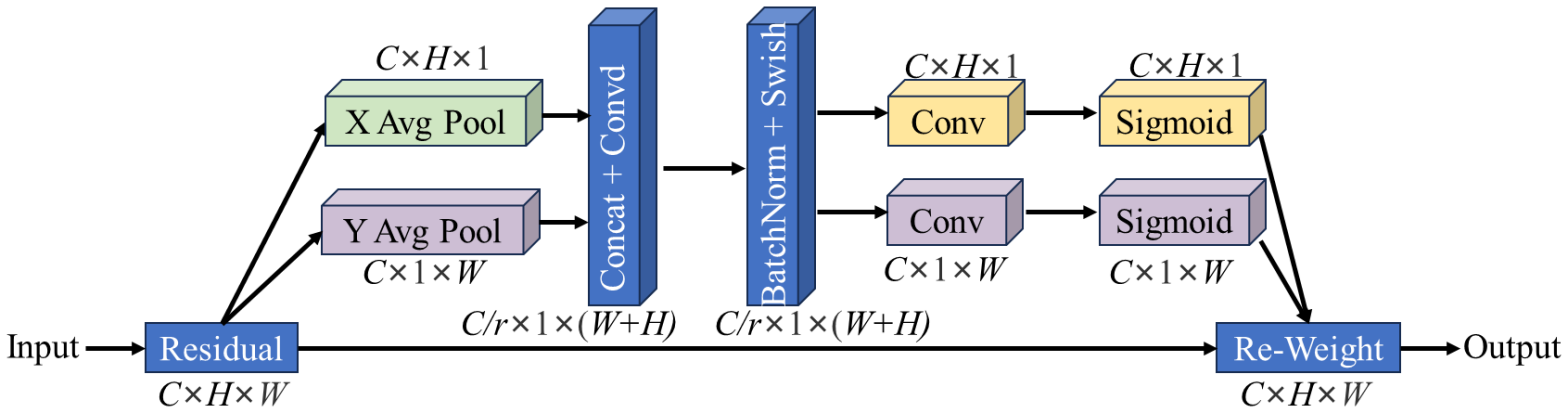
The structure of CA modules. “X/Y Avg Pool” is the average pool in X/Y direction; “Concat” stands for concatenate; “BatchNorm” stands for batch normalization; “Swish” and “Sigmoid” represent nonlinear activation functions; C is the number of channels; *H* is the height of the feature map; *W* is the width of the feature map; *R* is the reduction factor.

The CA module can focus the model’s attention on the region of interest through effective positioning in the pixel coordinate system, thereby obtaining information that considers both channel and position in the tea leaf image, reducing the attention to interference information, and thus improving the feature expression ability of the model. The basic structure of the CA module is shown in **Figure 5**.

For a given characteristic graph *X*, the number of channels is *C*, the height is *H*, and the width is *W*. The CA module first pools the input X in two spatial directions, namely, height and width, to obtain feature maps in both directions. Next, it concatenates the feature maps from these two directions in spatial dimensions, and then changes the dimensions to the original *C/r* using a 1×1 convolution transformation. Subsequently, it applies batch normalization and Swish activation operations to obtain an intermediate feature map containing information from both directions, as shown in the formula below.


(1) $$f=\delta \left(F_{1}\left(\left[\frac{1}{W}\sum_{0\leq j\leq W}^{\infty }x_{c}\left(h,j\right),\;\frac{1}{H}\sum_{0\leq i\leq H}^{\infty }x_{c}\left(i,w\right)\right]\right)\right)$$

In the formula, *f* is the intermediate feature map obtained by encoding spatial information in two directions, *δ* is the activation function Swish, and F1 is the convolution transformation function of 1×1. Here, *x_c_* is the feature information of the specific position of the feature graph in channel c, *h* is the specific height of the feature map, and *j* is the width of the feature map, with the value range of [0, W]. Similarly, *w* is the specific width of the feature map, and *i* is the height of the feature map, with the value range of [0, H]. F is decomposed into two separate tensors *h^f^* and *w^f^* along the spatial dimension in two directions. Through two 1×1 convolution transformation functions, *h^f^* and *w^f^* are converted into tensors with the same number of channels as the input *X*. Next, the attention weights in height and width are obtained by activating the function σ. Finally, we multiply the extended attention weight with *X* to get the output of the CA module, as shown in the equation below.


(2) $$y_{c}=x_{c}\left(i,j\right)\cdot \left(\sigma \left[F_{h}\left(f^{h}\right)\right]\right)\cdot \left(\text{σ}\left[F_{w}\left(f^{w}\right)\right]\right)$$

Where *y_c_* is the output of the c-th channel, σ is the activation function Sigmoid, and *F_h_* and *F_w_* are convolution transformation functions in height and width.

### 2.4 Transfer learning

In deep learning models, a large number of parameters are typically required for training, often necessitating extensive support from large-scale datasets. However, not all tasks have access to sufficiently large datasets for training. Transfer learning offers a solution to this issue. Transfer learning facilitates the application of the same model to another research context. Given that there are often commonalities between different samples, sharing similar characteristics, leveraging models pre-trained on large datasets to retrain for new tasks can achieve effective training outcomes and rapid convergence speeds. Therefore, in order to make full use of the existing labeled data and ensure the recognition accuracy of the model on new tasks, this study adopts transfer learning to optimize the model. Specifically, the fine-tuning method involves freezing part of the convolution layers as the optimization strategy for transfer learning. First of all, the large dataset ImageNet (Szegedy et al., 2015) serves as the source domain for network pre-training. The learned model weights from this pre-training phase are then transferred to identify diseases and insects in tea leaves. Drawing on existing prior knowledge allows for efficient handling of similar recognition tasks. Subsequently, the model parameters are fine-tuned during the training process on images of tea leaf diseases and insects, ultimately producing the final tea leaf disease and insect recognition model.

## 3 Results and discussion

### 3.1 Test environment and parameter setting

To evaluate the performance of the tea leaf disease and insect recognition model MobileNetV3-CA, acquired images of tea leaf diseases and insects were used for both training and testing. The dataset was divided into training, validation, and test sets in a ratio of 7:2:1. The experiment employed the PyTorch 1.10.0 deep learning framework, programmed in Python 3.8. The development environment was set up using VSCode. The computer used for running the programs is equipped with an Intel^®^ Core i5-1135G7 CPU @ 2.40 GHz, 32 GB of RAM, and operates on a 64-bit Windows 10 system.

The Batch Size of the experiment was set to 16. In order to improve the convergence of the model, the classified cross-entropy is used as the loss function, and the random gradient descent method (Stochastic Gradient Descent, SGD) is used to train the model. The learning rate, weight attenuation and momentum of the three training parameters are set to 0.001, 0.00001 and 0.9, respectively, and the learning rate attenuation strategy is set. Every 5 Epoch, the learning rate decays to 80% of the original.

### 3.2 Evaluation index

In order to comprehensively evaluate the performance of the MobileNetV3-CA model, this experiment selected four indicators to comprehensively evaluate the recognition effect of the model: Precision, Recall, F1-score and Accuracy. The calculation formulas are as follows:


(3) $$Precision=\;\frac{TP}{TP+FP}\times 100\%$$

(4) $$Recall=\;\frac{TP}{TP+FN}\times 100\%$$

(5) $$F1\_score=\;2\times \frac{Recall\;\times Precision}{Recall+Precision}$$

(6) $$Accuracy=\;\frac{TP+TN}{TP+TN+FP+FN}\times 100\%$$

In the formula, TP, FP, FN and TN are the statistics of the classification of different tea insects and diseases by the classification model in the confusion matrix respectively. Among them, TP(True Positive) represents the number of samples whose true value is positive and identified as positive, FP(False Positive) represents the number of samples whose true value is negative but identified as positive, FN(False Negative) represents the number of samples whose true value is positive but identified as negative, and TN(True Negative) represents the number of samples whose true value is negative and identified as negative. For the purpose of identifying diseases and insects, the actual number of categories of samples to be identified is regarded as the positive sample number, while the sum of all other categories is considered the negative sample number.

### 3.3 Comparative experiment of transfer learning training methods

**Figure 6:**
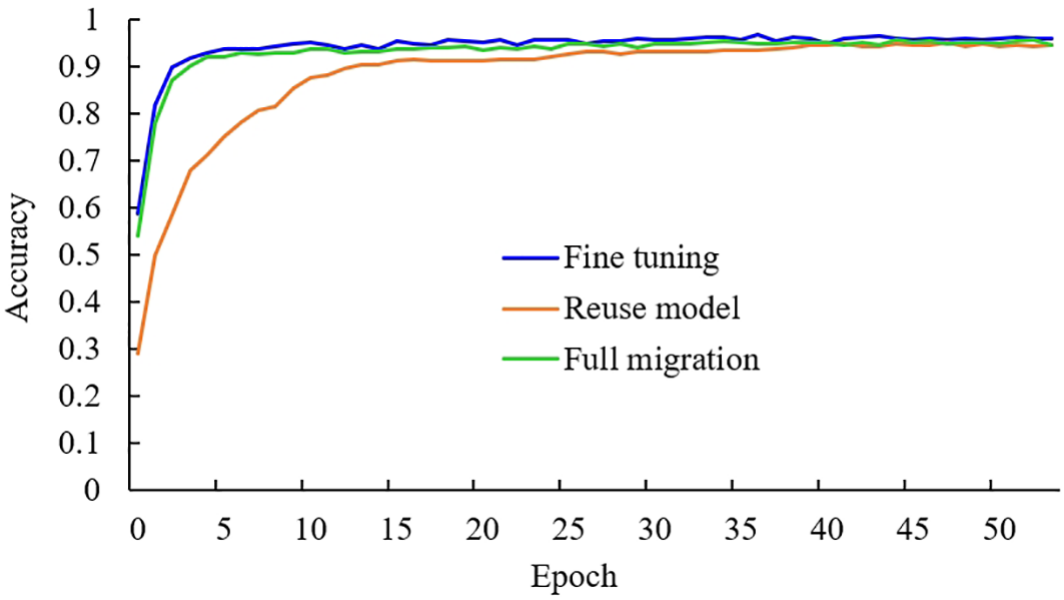
Accuracy variation curves of three transfer methods on validation set.

There are three common ways of transfer learning: the full parameter migration method, which involves freezing all convolution layers and only training the fully connected layer; the reuse model method, which only uses the model structure but not the pre-trained parameters; and the fine-tuning method, which involves freezing part of the convolution layers. This study adopts the fine-tuning method of freezing only part of the convolution layers. To test the effectiveness of the fine-tuning method used in this study, the above three transfer learning methods were used to train three models. Experiments were conducted based on the improved MobileNetV3-CA model using the same experimental data. The experimental results are shown in **Table 2**, and the variation curve of training and validation accuracy of the three transfer learning methods is shown in **Figure 6**.

**Table 2:** Performance of MobileNetV3-CA trained by 3 different transfer methods.

Methods	Precision /%	Recall /%	F1-score /%	Accuracy /%
Fine tuning	95.98	96.19	96.01	95.88
Full migration	94.66	93.96	94.33	94.14
Reuse model	94.89	94.55	94.72	94.58

It can be seen from **Table 2** that under the same experimental conditions, the accuracy of the fine-tuning method is the highest, while the accuracy of the full migration method is the lowest. Additionally, the F1 value of the fine-tuning method is also the highest among the three methods. In the full parameter migration method, all the initial parameters of the model are obtained by pre-training, and only the fully connected layer is trained, making it difficult to optimize the model’s parameters. As shown in **Figure 6**, in the reuse model method, the initial parameters of the model are set randomly, and it takes a long time to improve the recognition accuracy. In contrast, the fine-tuning method uses initial parameters obtained after extensive data training, rather than random settings, allowing the model to find suitable parameters more quickly. Moreover, the fine-tuning method retrains the convolution layers in the middle of the model, making the model parameters more suitable for the tea leaf disease and insect identification task. Therefore, the four indicators in the comprehensive experiment show that the fine-tuning transfer learning method used in this study is more effective than the other two transfer learning methods.

### 3.4 Performance analysis of MobileNetV3-CA model

**Figure 7:**
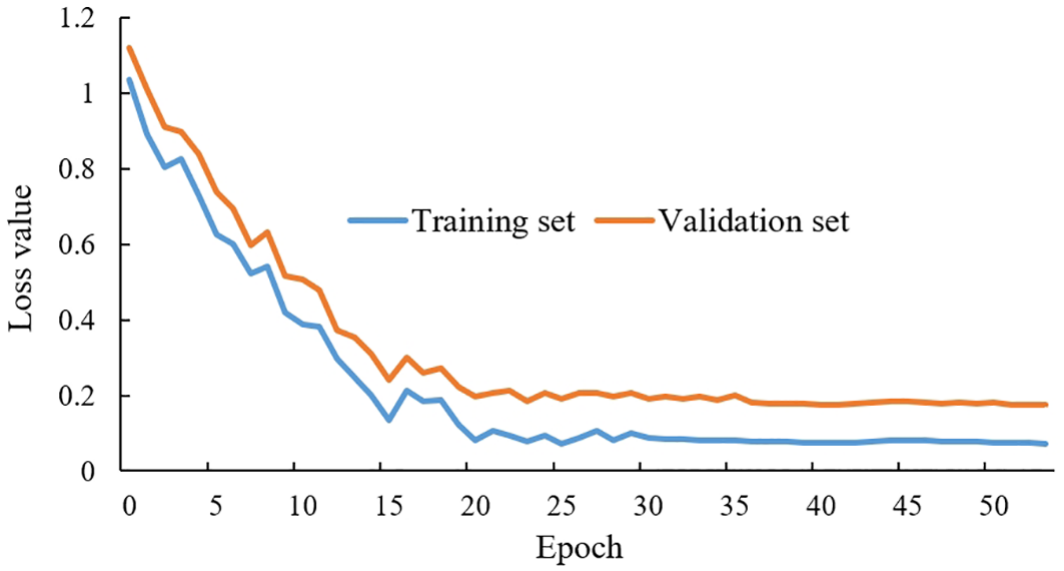
Loss value curves of the MobileNetV3-CA model on the training set and validation set.

In the fine-tuning transfer learning method, the loss value change curve of the MobileNetV3-CA model on the self-built training set is shown in **Figure 7**. During the training process, the model’s loss value decreased rapidly in the first 10 epochs and gradually slowed down after 10 epochs of training. By the time the training reached 20 epochs, the loss value curves of the model tended to flatten, indicating that the MobileNetV3-CA model had reached saturation. Notably, during the training process, the change trend of the loss curve of the MobileNetV3-CA model on both the training and validation sets was basically the same. This shows that the overall convergence trend of the model is good and there is no overfitting, verifying the effectiveness and learnability of the MobileNetV3-CA model.

In order to further verify the performance of the MobileNetV3-CA model, the classification results on the self-built test set were analyzed. The test set contained 17 species of tea leaf pests and diseases, including 200 pictures for each species, for a total of 3400 pictures. Overall, the average recognition accuracy, recall rate, and F1-score of the MobileNetV3-CA model on the test set are 95.88%, 96.19%, and 96.01%, respectively, all exceeding 95%. These experimental results demonstrate that the improved MobileNetV3-CA model can efficiently locate and extract small feature differences in tea leaf disease and insect images.

### 3.5 Comparative experiment on different attention mechanisms

In order to further verify the competitive advantage of introducing the CA module into the attention module, the SE attention module in the MobileNetV3-Large model was replaced by two classical attention mechanisms, namely, the ECA (Efficient Channel Attention) module (Wang et al., 2020) and the CBAM (Convolutional Block Attention Module) (Woo et al., 2018), under the same experimental conditions.

**Figure 8:**
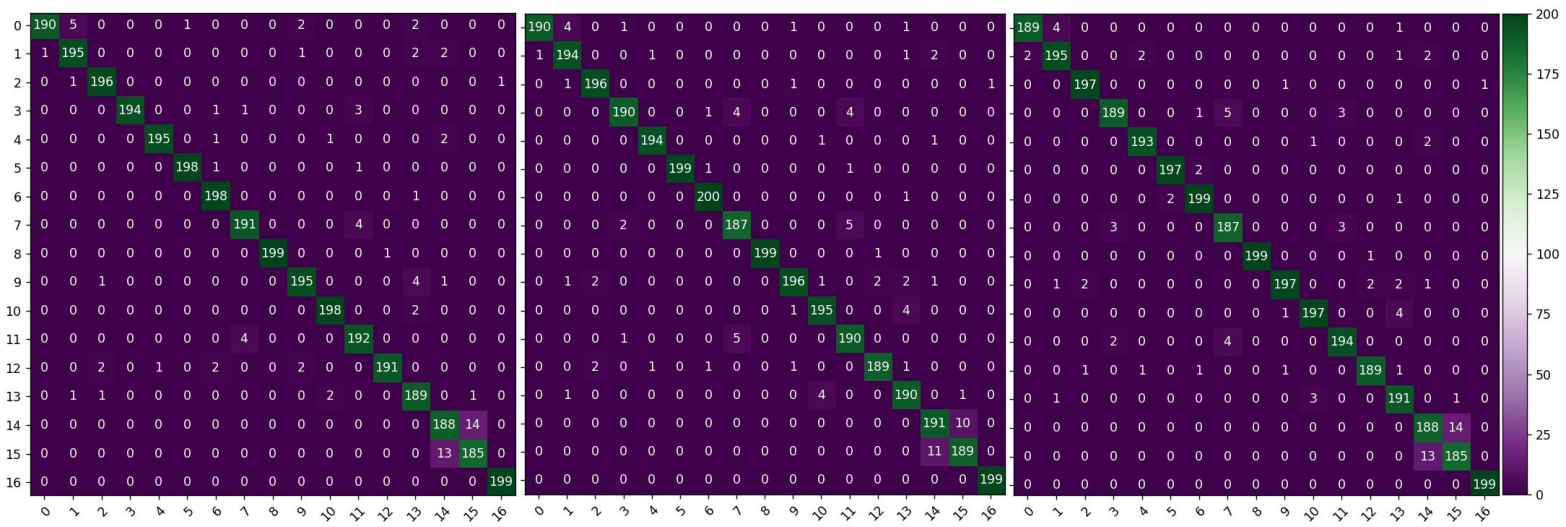
Confusion matrix of 3 different models.

**Figure 9:**
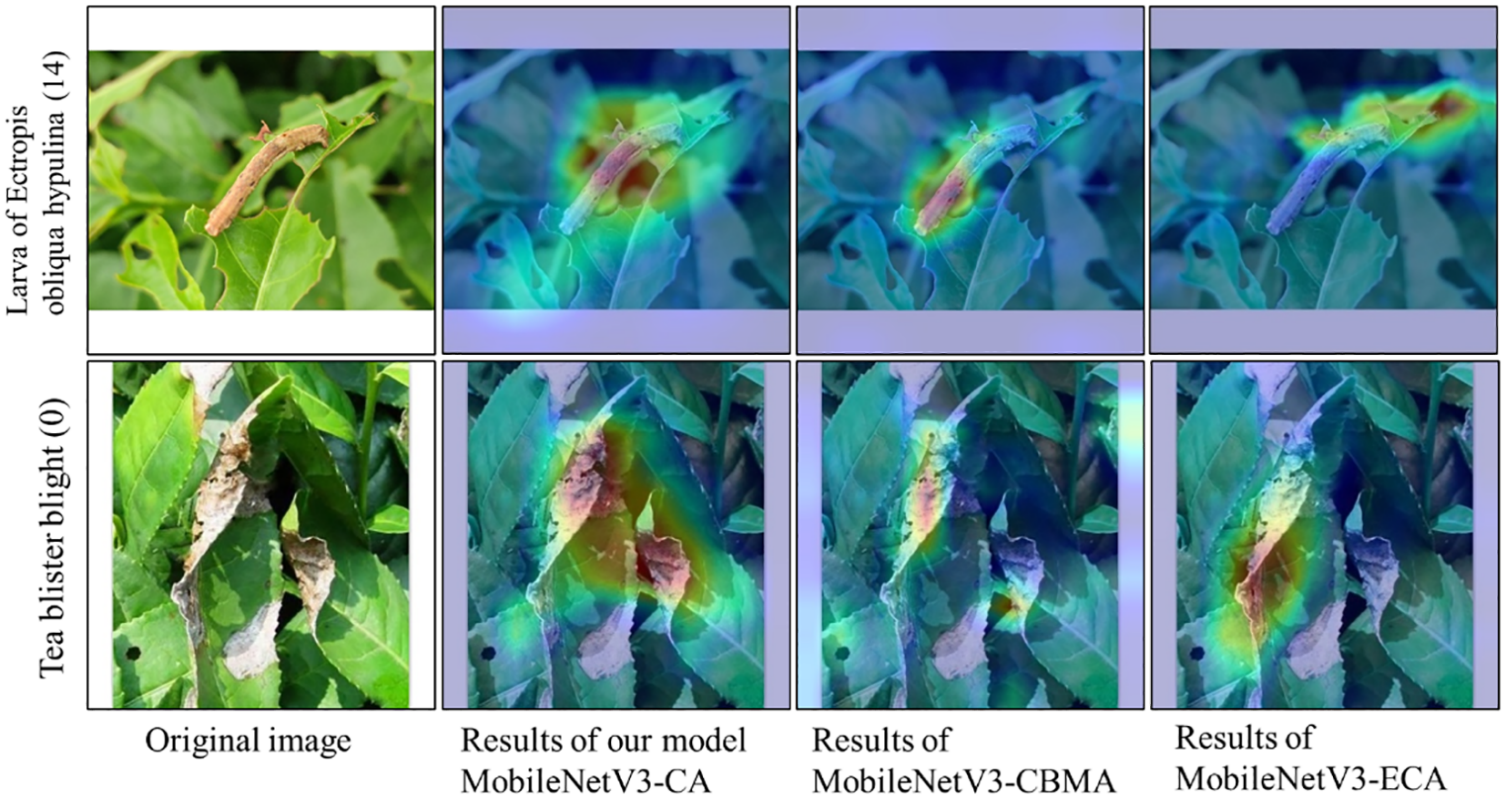
Feature visualization heat map of three different models.

**Figure 8** shows the confusion matrix of the recognition results for each model on the self-built test set. Overall, the recognition accuracy of the MobileNetV3-Large, MobileNetV3-CBAM, and MobileNetV3-CA models is 94.45%, 94.80%, and 95.88%, respectively. This demonstrates that compared with the other two models, the MobileNetV3-CA model can more accurately identify the characteristics of diseases and insects in tea leaves, effectively improving the model’s accuracy. Details in **Figure 9** show that the introduction of the ECA, CBAM, and CA modules can alleviate misclassification and omission issues in the MobileNetV3-Large model to some extent, making the model more suitable for identifying diseases and insects in tea leaves. Therefore, compared with other attention mechanisms, the introduction of the CA module can better improve the recognition performance of the MobileNetV3-Large model, verifying the competitive advantage of the CA module.

### 3.6 Comparative test of different models

Regarding image classification, ShuffleNetV2 and Inceptionv3 are two excellent lightweight convolutional neural networks that can be easily deployed on mobile devices. Meanwhile, AlexNet, VGG16, and the ResNet series models are also representative convolutional neural networks in visual tasks, achieving excellent results in visual classification tasks. They provide valuable reference points and comparability for the tea leaf disease and pest recognition method proposed in this study. To demonstrate the effectiveness of the proposed model, it was compared with six other models using the same experimental data and training strategy. The performance of these models on the test set is shown in **Table 3**.

**Table 3:** Comparison of accuracy, parameters and computation between models.

Models	Accuracy/%	Params/M	Precision/%	Recall/%	F1-score/%
ResNet18	94.55	1.12 × 10^7^	94.02	94.24	94.02
AlexNet	90.22	1.46 × 10^7^	90.45	90.66	90.87
VGG16	94.08	1.34 × 10^6^	93.55	93.42	94.46
SqueezeNet	88.40	7.25 × 10^5^	88.34	89.21	88.26
ShuffleNetV2	92.23	1.26 × 10^6^	92.29	92.33	92.22
MobileNetV3-large	94.45	4.21× 10^6^	94.96	94.29	94.12
MobileNetV3-CA	95.88	2.7 × 10^6^	95.98	96.19	96.01

As seen in the table, ResNet18 has strong feature extraction ability and shows good recognition performance in the experiment, but it consumes a lot of memory and computing resources. The accuracies of AlexNet and VGG16 models on the test set are slightly lower than that of the ResNet18 model. Additionally, both models have a large amount of model parameters, which require more storage space. The SqueezeNet model has small parameters, but its accuracy index is low compared to other models. The ShuffleNetV2 model uses the idea of grouped convolution to reduce the number of parameters and calculations. However, its recognition accuracy is slightly poor on the tea leaf diseases and insects image dataset with insignificant feature differences, resulting in poor model stability. Although the MobileNetV3-Large model uses deep separable convolution to restrict the depth and width of the network, it still achieves excellent results in the task of disease and insect identification in tea leaves, with performance close to ResNet18 and better than ShuffleNetV2, AlexNet and SqueezeNet. Compared with other models, the MobileNetV3-CA model achieves better recognition results, with a recognition accuracy as high as 95.88%, which is 1.33, 5.66, 1.80, 7.48, 3.65, and 1.43 percentage higher than ResNet18, AlexNet, VGG16, SqueezeNet, ShuffleNetV2, and MobileNetV3-Large, respectively. In general, the MobileNetV3-CA model not only ensures the detection speed but also improves the identification efficiency of diseases and pests in tea leaves, better balancing the complexity and recognition effect of the model.

### 3.7 Application of the proposed recognition model

**Figure 10:**
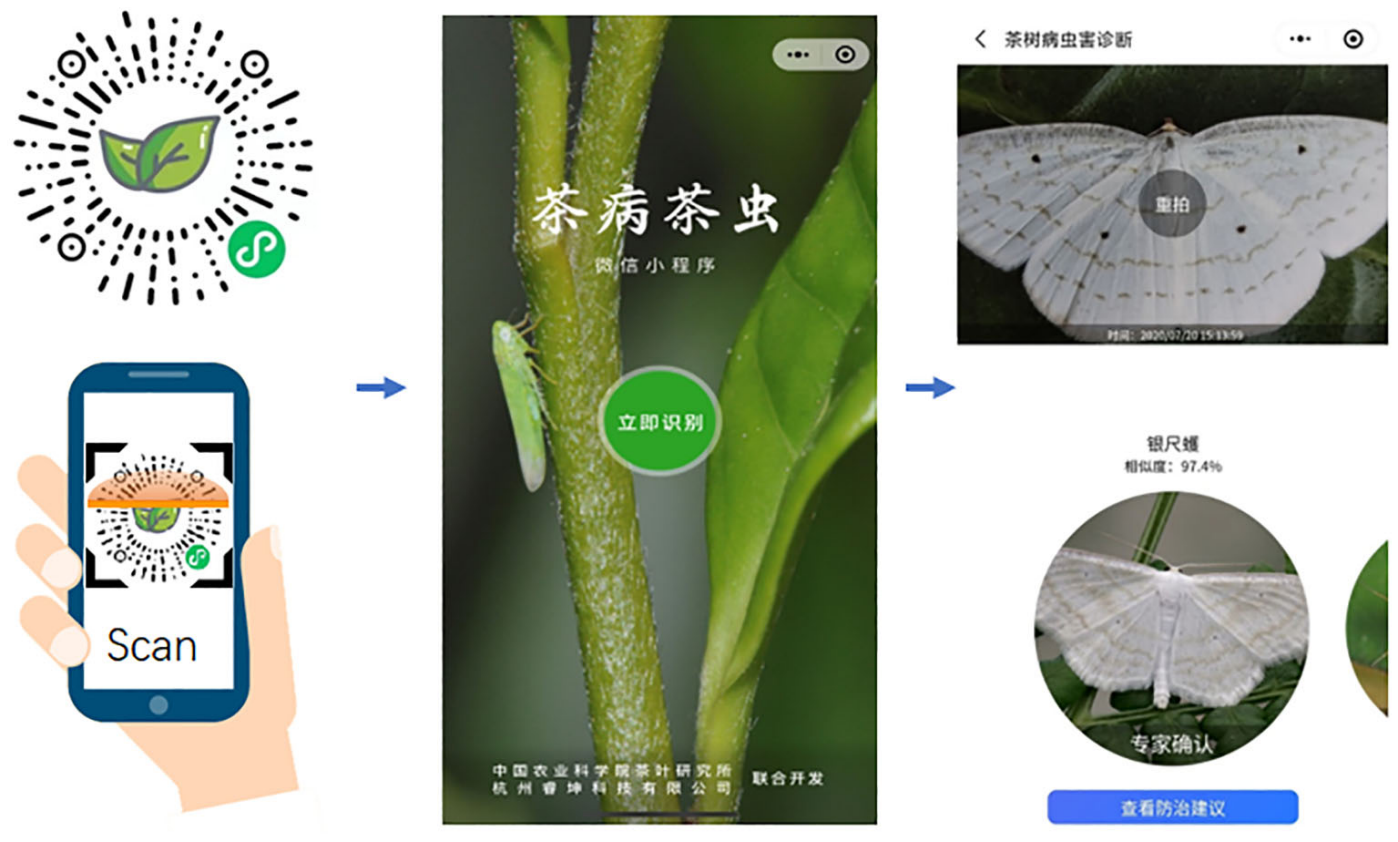
The interface of the mobile recognition process and results of the recognition model.

To verify the practical application effectiveness of this method and to better support actual tea production, an application program for identifying tea diseases and insects was developed using cloud and mobile terminals. Users can capture or upload images of tea leaves with diseases or insects using their mobile devices, which are then sent to the cloud for processing. The cloud-based program identifies tea leaf insects and diseases using the developed identification model and sends the identification results back to the mobile terminal. The interface of the mobile recognition process and results can be seen in **Figure 10**. The recognition results include the most probable category information of diseases and insects, such as the category name and the probability of the category label, accompanied by corresponding prevention and control suggestions.

The system has been implemented in tea gardens located in Xinchang County, Zhejiang Province, and Fuding County, Fujian Province, China. It has been utilized to identify and diagnose numerous common tea leaf diseases and insects, such as tea blister blight, tea anthracnose, larva of Ectropis obliqua hypulina, Chrysopa sinica, among others, achieving an average accuracy of 90.36%. According to the test results, due to the similarities among certain diseases and insects, occasional misjudgments may occur; however, the average misjudgment rate remains below 6%. Hence, this system holds practical value for application.

## 4 Conclusion

In summary, this study makes several significant contributions. It introduces an enhanced classification algorithm for tea leaf disease and insect recognition based on MobileNetV3, leveraging the CA attention mechanism and transfer learning to notably improve model performance.

1. The research presented an improved classification algorithm for tea leaf disease and insect recognition, named MobilenetV3-CA, which builds upon the MobileNetV3 architecture. The findings demonstrate that incorporating the CA attention mechanism into the MobileNetV3 model could enhances the performance of disease and insect recognition in tea leaves. The introduction of the CA attention mechanism enables the model to better comprehend and utilize spatial information, thereby improving its ability to identify disease locations.

2. The study also investigates the algorithm’s effectiveness in transfer learning, validating its ability to improve model performance. Through transfer learning, the model can rapidly adapt and learn when faced with new tea leaf disease data, and the accuracy rate of disease recognition is raised from 94.45% to 95.88%, resulting in an overall increase in recognition accuracy. This provides a reliable theoretical basis and experimental support for the application of the MobilenetV3-CA algorithm in actual tea leaf disease monitoring systems.

3. The advancements in this study offer promising applications in real-world tea leaf disease monitoring and management systems, introducing innovative approaches for integrating intelligent technologies into agriculture. However, the current images of tea tree leaf pests primarily focus on the adult stage of the pests, a point at which the infestation may have already spread extensively or become significantly harmful. The next step is to collect more images of pests in their earlier stages to better facilitate early identification and warning of pest outbreaks. Additionally, future research directions may include further extending the application of this algorithm to the identification of other stages of insect pests and to other areas of crop pests and diseases, thereby expanding its impact and utility in agricultural practice.

## Data Availability

The raw data supporting the conclusions of this article will be made available by the authors, without undue reservation.
